# Scanning tunneling microscopy and spectroscopy of rubrene on clean and graphene-covered metal surfaces

**DOI:** 10.3762/bjnano.11.100

**Published:** 2020-08-03

**Authors:** Karl Rothe, Alexander Mehler, Nicolas Néel, Jörg Kröger

**Affiliations:** 1 Institut für Physik, Technische Universität Ilmenau, D-98693 Ilmenau, Germany

**Keywords:** graphene, metal surfaces, molecular superstructures, rubrene, scanning tunneling microscopy, scanning tunneling spectroscopy, vibronic states

## Abstract

Rubrene (C_42_H_28_) was adsorbed with submonolayer coverage on Pt(111), Au(111), and graphene-covered Pt(111). Adsorption phases and vibronic properties of C_42_H_28_ consistently reflect the progressive reduction of the molecule–substrate hybridization. Separate C_42_H_28_ clusters are observed on Pt(111) as well as broad molecular resonances. On Au(111) and graphene-covered Pt(111) compact molecular islands with similar unit cells of the superstructure characterize the adsorption phase. The highest occupied molecular orbital of C_42_H_28_ on Au(111) exhibits weak vibronic progression while unoccupied molecular resonances appear with a broad line shape. In contrast, vibronic subbands are present for both frontier orbitals of C_42_H_28_ on graphene. They are due to different molecular vibrational quanta with distinct Huang–Rhys factors.

## Introduction

Two-dimensional materials are emerging as monatomically thin buffer layers (BLs) on metal surfaces, i.e., as intermediate films that efficiently reduce the hybridization of an adsorbate with the metallic substrate. The minimization of the adsorbate–substrate coupling is motivated by the desire to preserve genuine properties of the free atom or molecule even after adsorption. For instance, adsorption on a BL often retains the sharp electronic and vibrational energy levels that are characteristic for the atom or molecule vacuum state and that would inevitably be broadened or even quenched upon adsorption on the metal surface. Narrow molecular resonances on surfaces are desirable because they increase the residence time of injected charge at the adsorbate, which is favorable for, e.g., energy conversion processes or the observation of vibronic progression [1].

Structural aspects of adsorption on the prominent two-dimensional materials graphene [2] and hexagonal boron nitride (h-BN) [3] have been studied in detail. In contrast, vibrational spectroscopy at the single-molecule level is scarce. Scanning tunneling spectroscopy (STS) of vibronic levels of 1,3,5-tris(2,2-dicyanovinyl)benzene on graphene-covered h-BN on SiO_2_ [4], of cobalt phthalocyanine molecules on graphene-covered SiO_2_/Si samples [5] as well as on h-BN-covered Ir(111) [6], of conjugated oligohenylenes on h-BN-covered Cu(111) [7], of manganese phthalocyanine on h-BN-covered Rh(111) [8], and of 5,10,15,20-tetraphenylbisbenz[5,6]indendo[1,2,3-*cd*:1′,2′,3′-*lm*]perylene on graphene-covered Ir(111) [9] have been reported so far. In these studies molecular orbitals, the highest occupied molecular orbital (HOMO) or the lowest unoccupied molecular orbital (LUMO), appear with spectroscopic fine structure in differential conductance (d*I*/d*V*, *I*: tunneling current, *V*: bias voltage) data, which is assigned to vibronic progression induced by a single group of molecular vibrations with similar quantum energies. Another type of two-dimensional materials is represented by single layers of transition metal dichalcogenides. A single layer of MoS_2_ on Au(111) has recently been used to electronically decouple 2,5-bis(3-dodecylthiophen-2-yl)thieno[3,2-*b*]thiophene from the metal surface [10]. A rich vibronic fine structure was observed in the HOMO spectroscopic signature induced by several fundamental vibrational modes of the molecule together with their higher harmonics and combination vibrations.

**Figure 1:**
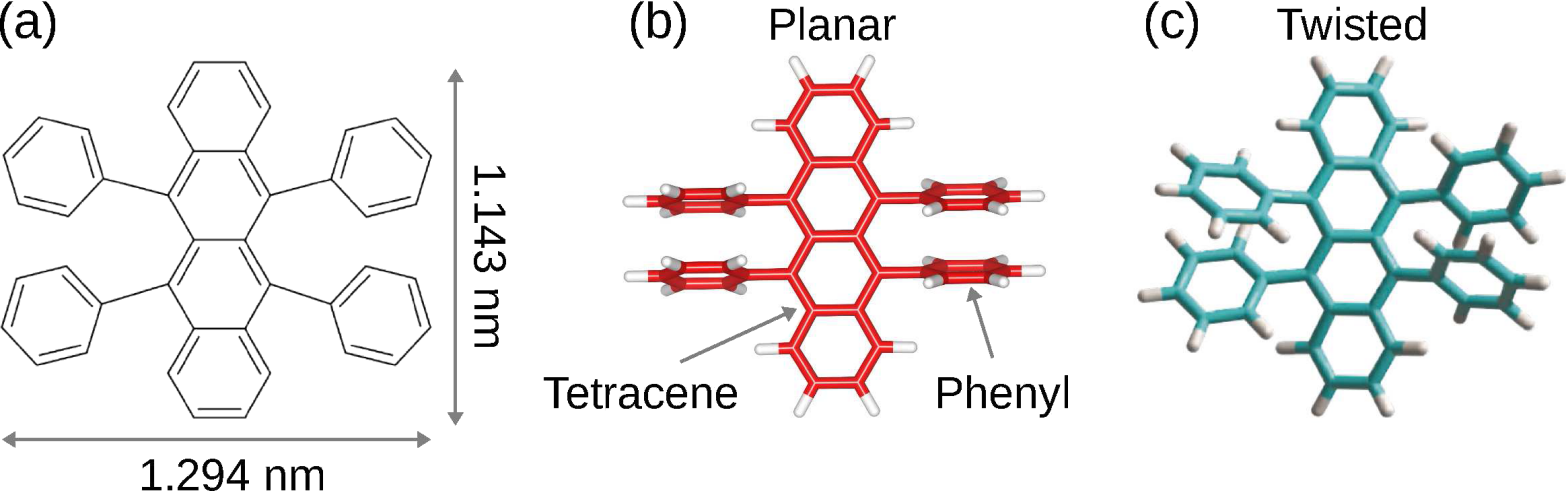
(a) Skeletal formula of C_42_H_28_ including dimensions. (b) Sketch of the planar molecular configuration. (c) Sketch of the twisted molecular configuration. Adapted with permission from [22], copyright (2008) American Chemical Society.

In the work presented here, 5,6,11,12-tetraphenyltetracene (rubrene, C_42_H_28_, Figure 1) was adsorbed on different surfaces, namely Pt(111), Au(111), and graphene on Pt(111), in order to demonstrate a gradual reduction of the C_42_H_28_–surface hybridization. The choice of the molecule and substrate surfaces was motivated as follows. C_42_H_28_ is a polycyclic aromatic hydrocarbon (Figure 1a) with an extended system of delocalized π electrons. In the gas phase, intramolecular steric hindrance [11–12] causes the phenyl groups to rotate around their σ bonds, out of the tetracene backbone plane (Figure 1b). The molecule therefore adopts a lander configuration that supposedly is beneficial to the electronic decoupling of its backbone from the substrate it is adsorbed to. Moreover, a twisted tetracene backbone (Figure 1c) is energetically more favorable than its planar, i.e., nontwisted, geometry (Figure 1b) for the vacuum state of C_42_H_28_ [13–14]. Upon adsorption of the molecule, the backbone may even be tilted with respect to the surface plane, i.e., it encloses a finite angle with the surface and, thus, deviates from a parallel adsorption [15]. From a more general point of view, C_42_H_28_ has gained in importance for its suitability in light-emitting diodes [16] and organic field-effect transistors [17]. As substrate surfaces, Pt(111) and Au(111) were chosen for their different electronic structure around the Fermi level. Pt(111) exhibits a high density of d states close to the Fermi energy (*E*_F_) [18–19], while Au(111) is characterized by a surface-projected gap of sp-derived electron states [20]. Graphene on Pt(111) exhibits a considerable distance of 330 pm from the metal surface [21], which implies a weak graphene–metal hybridization. Adsorbates on graphene-covered Pt(111) are therefore expected to be well decoupled from the metal substrate.

After adsorption of C_42_H_28_ on Pt(111) scanning tunneling microscopy (STM) images reveal the occurrence of separate molecular clusters and very broad molecular resonances in STS data, which is attributed to an elevated C_42_H_28_–Pt interaction. On Au(111) the observations are compatible with a reduced hybridization since compact molecular islands with a regular superstructure form and vibronic progression of the HOMO d*I*/d*V* spectroscopic signature is visible. Unoccupied molecular orbitals, however, appear as broad and merged resonances without indication of a vibronic fine structure. The molecular superstructure on graphene is similar to the assembly on Au(111), albeit with a lower molecule surface density, and d*I*/d*V* data exhibit vibronic progression in both frontier orbitals, which reflects the effective separation of C_42_H_28_ from the metal surface.

## Experimental

The experiments were performed with an STM operated in ultrahigh vacuum (10^−9^ Pa) and at low temperature (Pt(111) and graphene-covered Pt(111) at 5 K, Au(111) at 78 K). Pt(111) and Au(111) surfaces were cleaned by Ar^+^ ion bombardment and annealing. Graphene was epitaxially grown on Pt(111) by exposing the heated (1300 K) surface to the molecular precursor C_2_H_4_ (purity 99.9%) at a partial pressure of 10^−4^ Pa for 120 s [23]. C_42_H_28_ molecules were sublimated from a powder (purity 98%), deposited in a heated (500 K) W crucible and directed towards the sample surface at room temperature. Molecular coverages below the closed single molecular layer are estimated from STM images as the percentage of the covered surface area. All STM images were recorded at constant current with the bias voltage applied to the sample. Constant-height d*I*/d*V* data were acquired with a lock-in amplifier by sinusoidally modulating the bias voltage (5 mV_rms_, 750 Hz) and measuring the first harmonic of the current response of the tunneling barrier.

## Results and Discussion

### Pt(111)–C_42_H_28_

**Figure 2:**
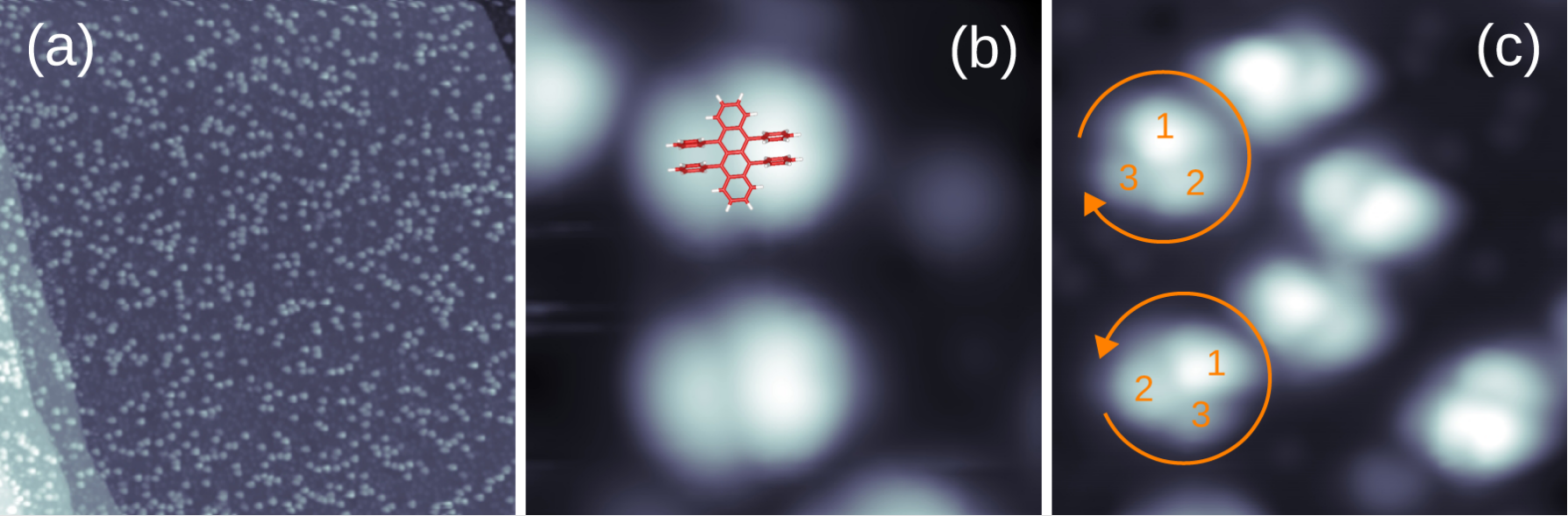
(a) STM image of C_42_H_28_ molecules on Pt(111) (bias voltage: 1 V, tunneling current: 100 pA, size: 150 × 150 nm^2^). (b) Close-up view of C_42_H_28_ with twisted configuration of the backbone 1 V, 100 pA, 5 × 5 nm^2^). The attached molecule sketch is to scale. (c) Close-up view of C_42_H_28_ with twisted and tilted configuration of the backbone (1 V, 100 pA, 7.1 × 7.1 nm^2^). **1**, **2**, and **3** number the brightest to the dimmest lobe and reveal the two rotational senses.

Figure 2a shows an overview STM image of C_42_H_28_ on Pt(111) at a coverage of approx. 20%. Well separated molecules or clusters of molecules can be discerned on the terrace without evidence for island formation or decoration of substrate step edges. These observations are indicative of an effectively reduced surface diffusion after adsorption, which lowers the mobility of adsorbed molecules and, thus, the formation of large molecular islands. The close-up view in Figure 2b shows that C_42_H_28_ molecules exhibit a submolecular structure consisting of two bright lobes separated by a central line with dim contrast. Some molecules even exhibit three lobes (Figure 2c). The two bright lobes of C_42_H_28_ in Figure 2b are assigned to one pair of phenyl groups each, while the dim line is attributed to the tetracene backbone (Figure 1). The two opposite pairs of phenyl groups appear with different contrast. Considering geometric properties alone, these microscopic observations contradict the twisted configuration of the molecule (Figure 1c) where one diagonal pair of phenyl groups would appear higher than the other. Such a configuration was indeed observed for a closed monolayer of C_42_H_28_ on Ag(100) [24]. However, it is difficult to infer geometric heights from STM images due to variations in the local density of states. Moreover, the elevated hybridization of C_42_H_28_ with Pt(111) is likely to distort the relaxed vacuum configuration of the molecule. A comparison of experimental data with simulated STM images would allow for further insight into the actual adsorption geometry; however, this is out of the scope of this work. The absence of individually resolved C_42_H_28_ phenyl groups on Pt(111) may be ascribed to the aforementioned elevated hybridization of the molecular and the substrate electronic structure, possibly mediated by the high density of Pt d bands at *E*_F_ [18–19]. In such cases, submolecular motifs are often suppressed.

Before corroborating the suggestion of elevated molecule–substrate coupling by STS data the occurrence of chiral molecular species shall be discussed. The close-up STM image in Figure 2c shows adsorbed C_42_H_28_ molecules that appear with three rather than two lobes. Moreover, numbering the brightest to the dimmest lobe with **1**, **2**, and **3** reveals that two senses of rotation, clockwise (*R*) and anticlockwise (*L*), occur. In agreement with previous findings for C_42_H_28_ on Au(111) [25–26], the three lobes reflect a tilted molecular backbone. Lobes **1** and **2** are assigned to one pair of phenyl groups each, similar to the assignment for molecules appearing with two lobes only. Lobe **3** is due to one end of the tetracene backbone that likely adopts an inclined adsorption geometry, i.e., encloses a finite angle with the Pt(111) surface plane. In the case of C_42_H_28_ on Au(111) this angle was determined as approx. 38° by near-edge X-ray absorption fine structure spectroscopy [15]. The observed chirality, *R*-C_42_H_28_ and *L*-C_42_H_28_, therefore results from the left or right pair of phenyl groups of a twisted C_42_H_28_ molecule being closer to the surface. On Au(111) the formation of homochiral clusters led to remarkable supramolecular assemblies [25–27], which, however, are not present on Pt(111).

**Figure 3:**
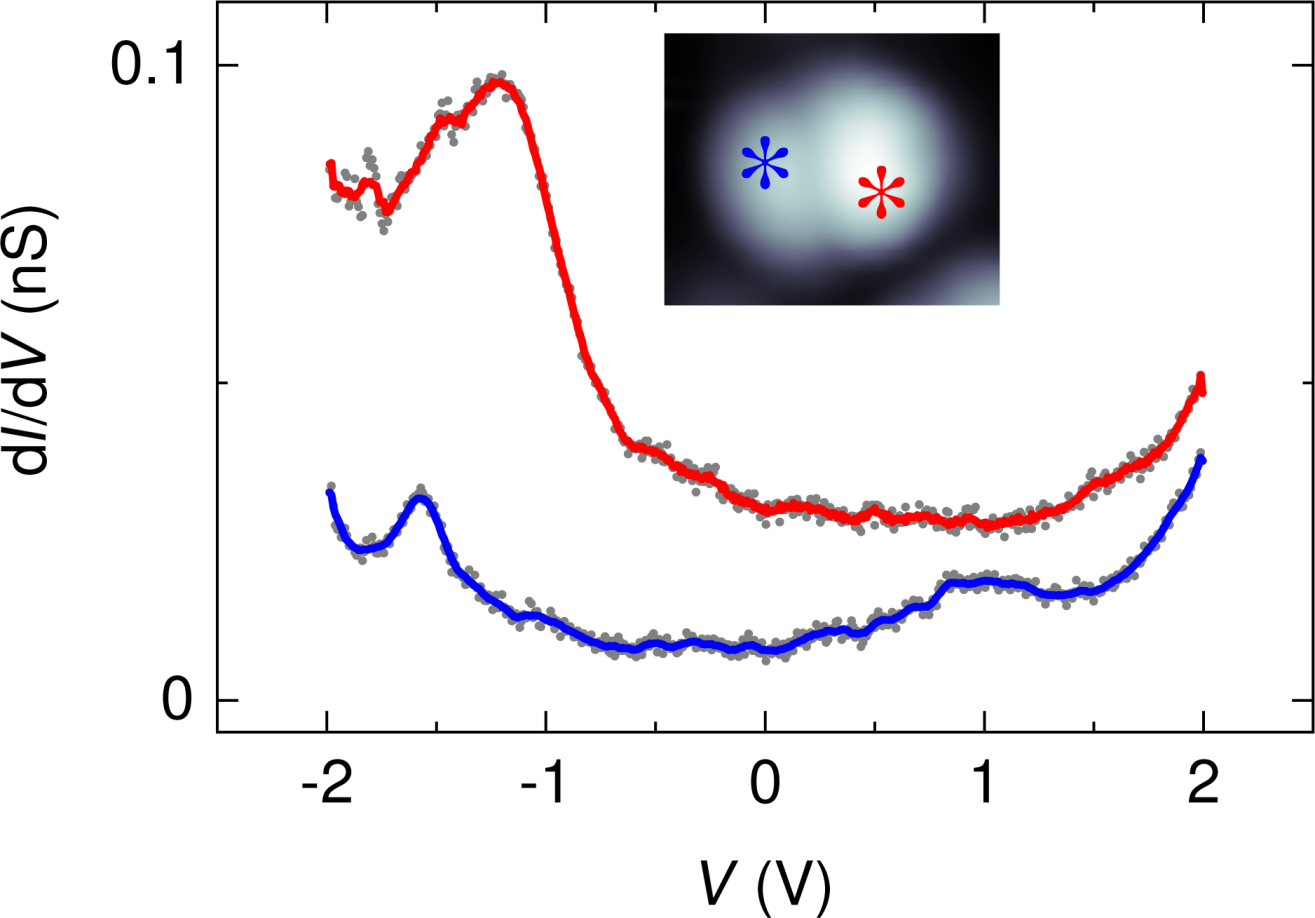
Spectra of d*I*/d*V* (dots) recorded above the different lobes of C_42_H_28_ on Pt(111) (feedback loop parameters prior to spectroscopy: 2.5 V, 100 pA). The solid lines represent smoothed data. The bottom (top) spectrum was acquired atop the left (right) lobe of the molecule as indicated in the inset. Inset: STM image of a single C_42_H_28_ molecule on Pt(111) (1 V, 100 pA, 3 × 2.5 nm^2^) with asterisks marking the positions of spectroscopy.

The presumably elevated C_42_H_28_–Pt(111) hybridization is corroborated by d*I*/d*V* spectra of the molecule. Figure 3 shows spectra recorded above intramolecular sites that approximately correspond to one phenyl group of the opposite lobes. The bottom spectrum was acquired atop the dim lobe of the molecule showing a peak at approx. −1.6 V and a rather broad feature at approx. 1.0 V. In contrast, d*I*/d*V* spectra recorded atop the bright lobe (top spectrum in Figure 3) only exhibit a broad peak at approx. −1.3 V and no further peak-like signature up to 2 V. Both spectra do not exhibit a clear-cut gap region, i.e., a bias voltage range with nearly vanishing d*I*/d*V* signal. These observations reflect the strong hybridization of C_42_H_28_ with the Pt(111) surface and hamper the meaningful determination of a HOMO–LUMO gap width. Moreover, the spectra of Figure 3 emphasize that a bright (dim) contrast does not necessarily imply a high (low) geometrical distance. The bright lobe of the molecule exhibits a d*I*/d*V* spectrum with a single broad orbital feature, which indicates its elevated hybridization with the metal surface rather than its greater distance from the surface.

### Au(111)–C_42_H_28_

**Figure 4:**
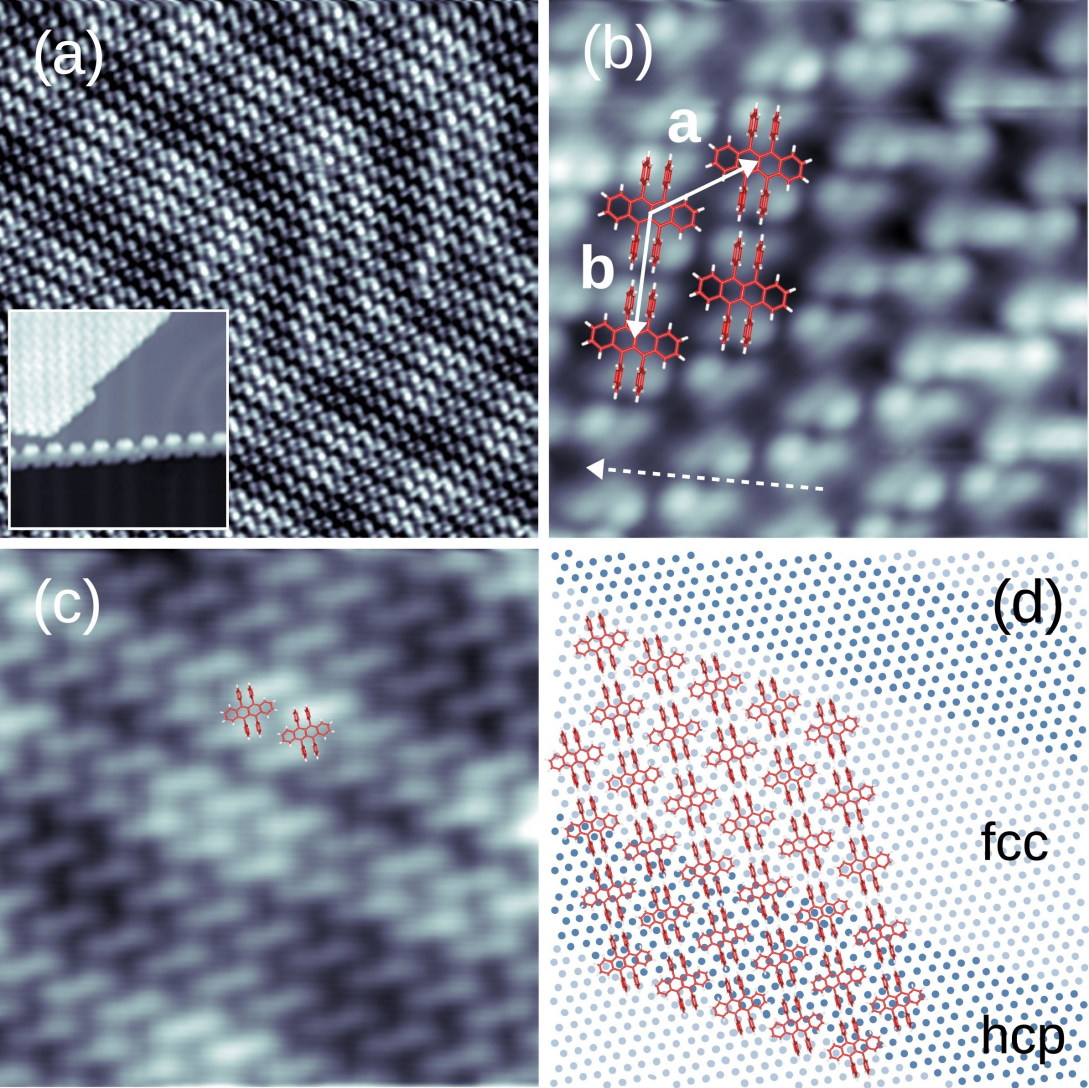
(a) STM image of C_42_H_28_ molecules on Au(111) (−0.8 V, 50 pA, 30 × 30 nm^2^). Inset: STM image of a Au(111) step edge decorated in a zig-zag manner with C_42_H_28_ molecules on the top and bottom terrace (−2 V, 50 pA, 19 × 19 nm^2^). (b) Close-up view of a molecular island (−0.65 V, 100 pA, 5 × 5 nm^2^). Sketches of C_42_H_28_ (to scale) are superimposed to facilitate the identification of individual molecules. The unit cell vectors **a** and **b** of the C_42_H_28_ lattice are indicated as arrows. The dashed arrow marks a 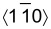 compact direction of Au(111). (c) Close-up STM image, which emphasizes the Au(111) reconstruction lines together with an additional periodic modulation of the apparent height along these lines (1 V, 50 pA, 10 × 10 nm^2^). (d) Sketch of suggested C_42_H_28_ adsorption on Au(111) with molecular rows being aligned with the separation of the fcc (bright dots) and hcp (dark dots) stacking domains of the surface reconstruction.

Figure 4a shows an overview STM image of C_42_H_28_ on Au(111) at a surface coverage of approx. 50%. The molecules form well-ordered large islands. In addition to the regular arrangement of C_42_H_28_, The STM image reveals a periodic pattern of stripes, which are due to the 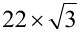 (or herringbone) reconstruction of Au(111) [28–30]. It consists of adjacent surface regions with alternating hexagonal close-packed (hcp) and face-centered cubic (fcc) stacking of Au(111) atom planes. These domains are separated by discommensuration lines (or soliton walls), which are visible as stripes with increased apparent height in Figure 4a. By measuring the width of hcp and fcc stacking domains on clean and C_42_H_28_-covered Au(111) we find that C_42_H_28_ adsorption leaves the surface reconstruction invariant. In addition, step edges of the Au(111) surface are decorated with molecules (inset to Figure 4a). These topographic data univocally hint at an enhanced C_42_H_28_ mobility after adsorption compared to the situation on Pt(111) (vide supra). The STM data presented in Figure 4a reveal an additional periodic modulation of the apparent height along the discommensuration lines, which will be discussed below.

The close-up STM image of a molecular island (Figure 4b) enables the resolution of individual molecules and the unit cell of the molecular superstructure. For negative bias voltages a single C_42_H_28_ molecule exhibits four lobes, which are attributed to the four phenyl groups. The superimposed molecule sketch visualizes this assignment. For some molecules the apparent height of all four phenyl groups is similar, while others exhibit a pair of diagonal phenyl groups appearing higher than the other pair, similar to previous findings on Ag(100) [24]. This observation is compatible with the presence of a mixture of molecules adsorbing with either a planar, i.e., all phenyl groups appear with similar heights, or a twisted, i.e., with different apparent heights for diagonal pairs of phenyl groups, tetracene backbone. The STM images do not indicate the presence of a tilted backbone. Therefore, the molecules adopt a nonchiral adsorption configuration, which contrasts the findings on Pt(111) (vide supra) and previously reported results for Au(111) [15,25–27]. In its crystalline phase the C_42_H_28_ molecule may indeed adopt the planar configuration of its tetracene backbone owing to a large cohesive energy that overcompensates through intermolecular hybridization the energy cost for planarization [13,31]. Flat C_42_H_28_ molecules were previously reported for monolayers on Au(111) [32], while on Bi(001) a coexistence of twisted and planar C_42_H_28_ was observed [33].

The unit cell of the superstructure is spanned by the lattice vectors **a** and **b** with 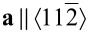 (crystallographic direction of soliton walls), |**a**| = 1.15 ± 0.10 nm, |**b**| = 1.30 ± 0.10 nm, 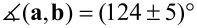, giving rise to a molecule surface density of approx. 0.67 nm^−2^. The tetracene backbone is oriented along a 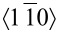 crystallographic direction of Au(111). In [32] a similar superstructure with planar C_42_H_28_ backbones oriented parallel to the Au(111) surface was reported. In that work a multilayer C_42_H_28_ film was deposited at room temperature and subsequently annealed to achieve a monolayer coverage. Different superstructures of C_42_H_28_ with twisted and tilted backbones were presented in [25–27,34] for Au(111) and in [35] for Bi(111). In these reports, the deposition of molecules was performed at 5 K [25–27,34] and 100 K [35] with subsequent annealing at room temperature [25–27,34] and 350 K [35]. Therefore, deposition at low temperature seems to favor the adsorption with twisted and tilted backbones, possibly due to an initial high density of small island with chiral molecules, while the subsequent annealing preserves the twisted and tilted configuration of the molecular backbone and leads to homochiral domains. The twisted configuration of the molecular backbone of C_42_H_28_ is not limited to low-temperature depositions. It was likewise observed for room-temperature deposition on low-index Cu surfaces [22,36] and Ag(100) [24].

The STM image in Figure 4c shows the influence of the 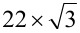 Au(111) reconstruction on the C_42_H_28_ assembly. Evidently, the molecular superstructure matches the period of the reconstruction, which is reflected by the alignment of molecule rows with the discommensuration lines. A single row of C_42_H_28_ occupies the top of the soliton walls and the narrow hcp stacking domain, while three rows of C_42_H_28_ reside atop the wider fcc surface regions (confer the sketch in Figure 4d for an illustration). Additionally, Figure 4c unveils a modulation of the apparent height along the discommensuration lines with a corrugation of approx. 9 pm, which is smaller than the approx. 26 pm modulation induced by the Au(111) reconstruction. As Figure 4d shows, nearly equivalent adsorption sites are occupied by the C_42_H_28_ molecules every 4**a** along the discommensuration lines. Therefore, the additional periodic pattern is due to a moiré effect along the soliton walls. A similar matching of the molecular superstructure with the Au(111) reconstruction was previously reported for C_64_H_36_ [37].

**Figure 5:**
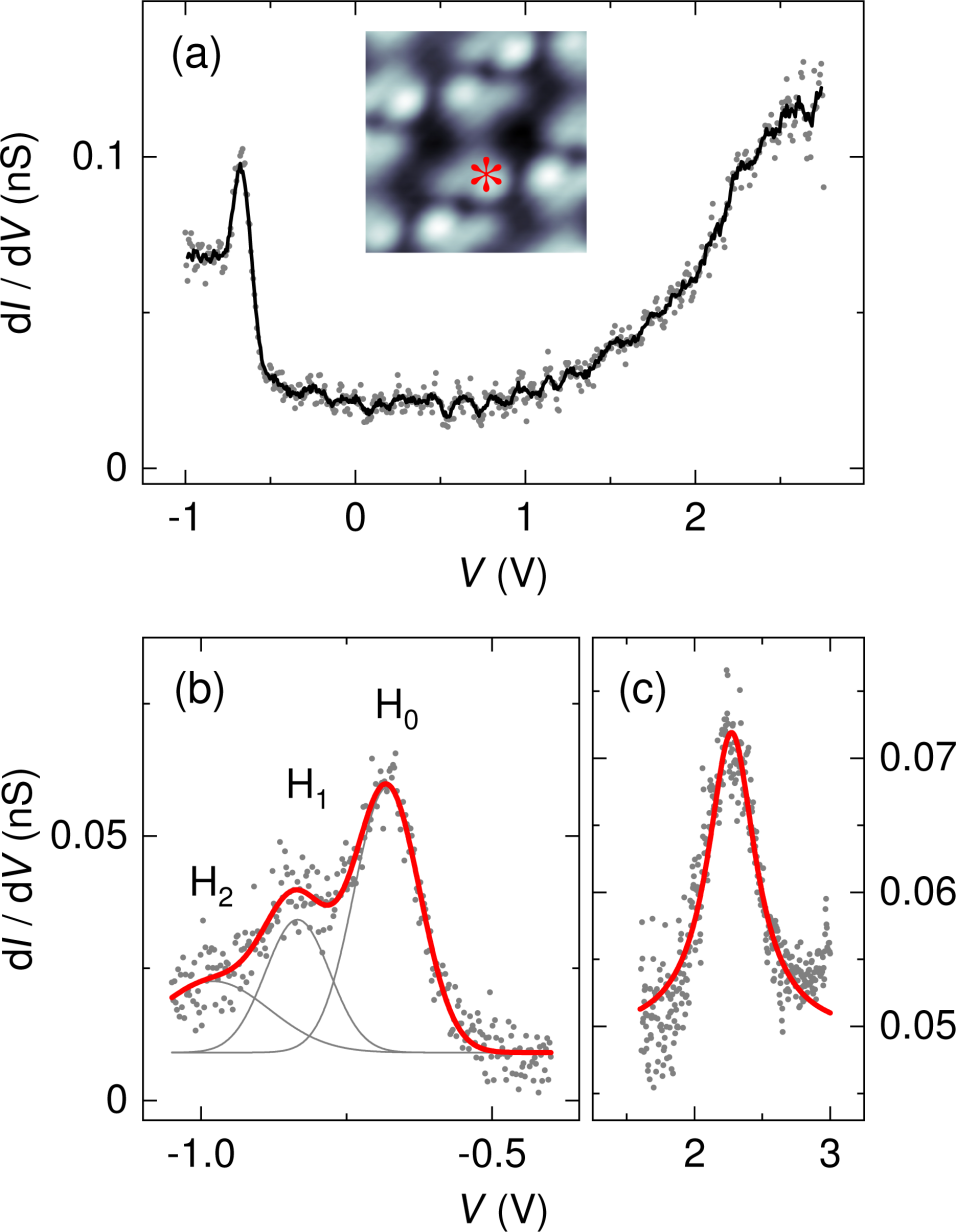
(a) Spectrum of d*I*/d*V* (dots) recorded above a C_42_H_28_ phenyl group on Au(111) with the spectroscopic signature of the HOMO appearing at approx. −0.67 V (feedback loop parameters: −1 V, 50 pA). The solid line represents smoothed data. Inset: STM image of a single C_42_H_28_ molecule on Au(111) (−0.65 V, 100 nA, 2 × 2 nm^2^) with the asterisk marking the position of spectroscopy. (b) Close-up view of the HOMO (H_0_) vibronic fine structure (feedback loop parameters: −1 V, 50 pA). The presented data (dots) are normalized [38]. Vibronic side bands are labeled H_1_ and H_2_. The thick solid line represents a fit of three Lorentzian line shapes (thin gray lines) and a constant background to the data. (c) Normalized d*I*/d*V* data (dots) showing a peaked unoccupied molecular orbital at approx. 2.3 V (feedback loop parameters: 3 V, 50 pA). The solid line represents the fit of a Lorentzian line shape and a constant background to the data.

Next, spectroscopic results obtained for C_42_H_28_ on Au(111) will be discussed. An overview spectrum is presented in Figure 5a where a peak-like signature is visible at approx. −0.67 V, which is assigned to the HOMO. The sharp line shape of the HOMO is in accordance with a theoretical work [34], which demonstrated a very low hybridization of the HOMO with the Au(111) electronic states. At positive bias voltage an increase of d*I*/d*V* for *V* ≥ 1 V is visible, probably due to the LUMO. Figure 5c shows a d*I*/d*V* spectrum in a higher bias voltage range of unoccupied states. The presented data were normalized by taking the exponential transmission factor of the tunneling barrier into account [38]. The normalized d*I*/d*V* data show a peak at approx. 2.3 V. Due to the considerably broad onset of d*I*/d*V* data starting from approx. 1 V it is difficult to unambiguously assign this peak to the LUMO or LUMO+1. Much wider unoccupied molecular resonances have been observed, too, in pump–probe photoemission experiments on thin C_42_H_28_ films adsorbed on highly oriented pyrolithic graphite and traced to the elevated molecule–substrate hybridization with a concomitant reduced lifetime of electrons injected into the LUMO [39]. Due to the absence of peaked orbital signatures a HOMO–LUMO gap width is hard to estimate. In a previous report, constant-current d*I*/d*V* data were presented for C_42_H_28_ on Au(111) and a HOMO–LUMO gap exceeding 3 eV was extracted [25]. A direct comparison to the spectroscopic data presented here is hampered due to the different spectroscopy mode applied and due to the presence of various molecular conformers [25] that were not observed in our experiments with a different preparation.

Using an increased bias voltage sampling in the HOMO spectral region exhibits d*I*/d*V* data with spectroscopic fine structure (Figure 5b). Three Lorentzian line shapes were superimposed to fit the normalized [38] data using a least-squares fit routine that adjusts the positions, widths and heights of the Lorentzian peaks. Aside from the peak labeled H_0_, which is attributed to the spectroscopic signature of the C_42_H_28_ HOMO, additional peaks H_1_ and H_2_ are observed. While the peak height of H_2_ is weak, its inclusion in the fit was necessary to closely describe the d*I*/d*V* data. The separations H_0_–H_1_ and H_1_–H_2_ are nearly identical, 160 ± 10 mV, which hints at vibronic progression due to a group of molecular vibrational modes with energies *h*ν ≈ 160 meV (*h*: Planck constant, ν: vibrational frequency). In vibronic progression the molecule is electronically and vibrationally excited from its ground state by an attached charge, i.e., hole or electron. Here, C_42_H_28_ is transiently charged by the extraction of an electron from the HOMO in the course of the tunneling process. Therefore, the peak labeled H_0_ in Figure 5 reflects the vibrational ground state of positively charged C_42_H_28_. If the energy of the tunneling electron is sufficiently large then vibrationally excited states of the transiently charged C_42_H_28_ may be reached, which appear as the satellite peaks H_1_ and H_2_ to H_0_. This resonant transition from a molecular ground state to an electronically and vibrationally excited state is described within the Franck–Condon picture [40]. In particular, the peak heights *I_ν,n_* of the vibronic subbands resulting from transitions of the vibrational ground state of neutral C_42_H_28_ to the *n*-th vibrationally excited and transiently charged C_42_H_28_ obey a Poisson distribution,

[1]
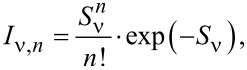


where *n* is the vibrational quantum number and *S*_ν_ is the Huang–Rhys factor, which determines the coupling of the hole or electron to the vibrational quantum with energy *h*ν. The Huang–Rhys factor can be expressed as

[2]
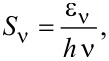


where ε_ν_ denotes the relaxation energy of the vibration when charging the molecule [10,41]. Comparing the peak height of the orbital signature (*I*_0_) with the first vibronic subband (*I_ν,1_*) enables direct access to the Huang–Rhys factor via

[3]
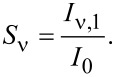


For the C_42_H_28_ HOMO on Au(111) a Huang–Rhys factor of approx. 0.5 can be extracted from d*I*/d*V* data (Figure 5b) for the vibrational mode with *h*ν ≈ 160 meV.

The energy of the vibrational mode is in good agreement with one of the three C_42_H_28_ vibrations that were previously determined on a theoretical basis to exhibit a particularly strong Holstein coupling to the HOMO of C_42_H_28_ embedded in a molecular crystal [42]. Generally speaking, the Holstein coupling describes the local interaction of a charge carrier with a molecular vibration [40] and may therefore be relevant to identify vibrational quanta that induce vibronic progression of a molecular orbital. In the particular case considered here, the charge carrier is a tunneling electron extracted from the C_42_H_28_ HOMO and may efficiently couple to the Holstein mode reported previously [42]. The calculated mode in question has an energy of 1349 cm^−1^ ≈ 167 meV and mainly consists of stretching vibrations of the tetracene skeleton where the HOMO is mostly localized [43–44].

### Graphene–C_42_H_28_

**Figure 6:**
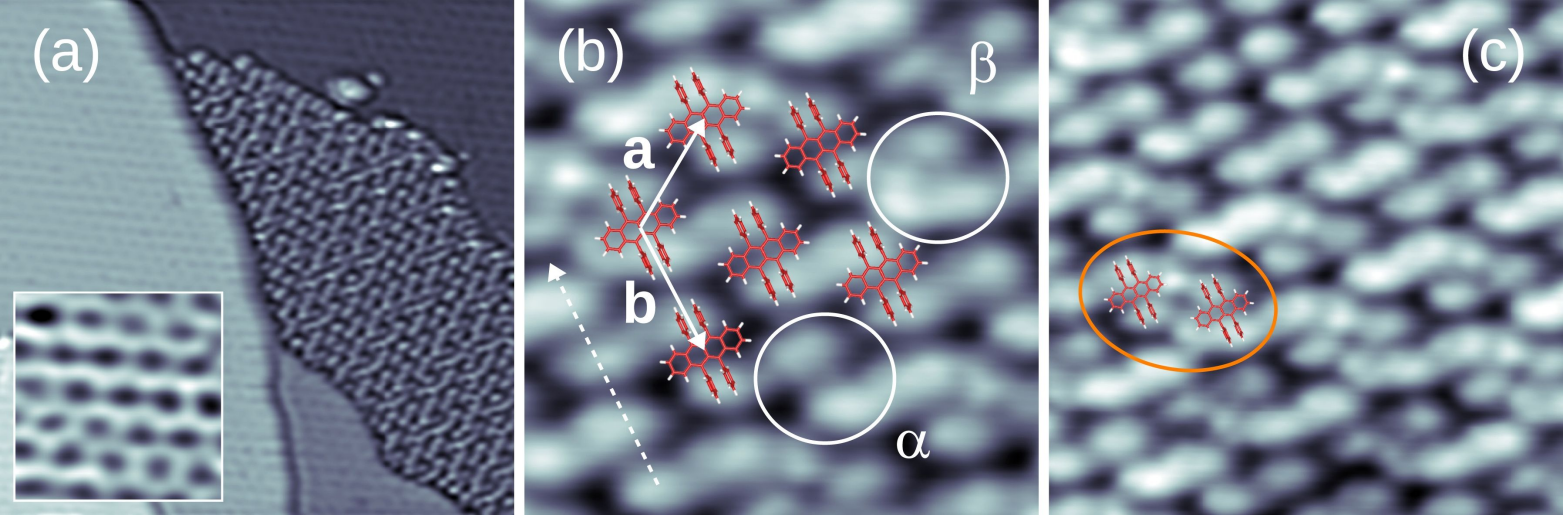
(a) STM image of C_42_H_28_ molecules on graphene-covered Pt(111) (2 V, 100 pA, 34 × 34 nm^2^). Inset: Close-up view of clean graphene with moiré pattern (5 × 5 nm^2^). (b) Close-up view of a molecular island (2 V, 20 pA, 6 × 6 nm^2^). Molecular sketches (to scale) of the C_42_H_28_ molecules are superimposed. The unit cell vectors **a** and **b** of the C_42_H_28_ lattice are indicated as arrows. The dashed arrow marks a 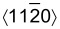 crystallographic direction of graphene. Molecules with planar (α) and twisted as well as tilted (β) configuration are encircled. (c) Close-up view of molecular rows of molecules with twisted and tilted configuration (2 V, 20 pA, 8.3 × 8.3 nm^2^). The ellipse indicates a pair of molecules with twisted and tilted configuration.

An STM image of C_42_H_28_ on graphene-covered Pt(111) at approx. 50% coverage is presented in Figure 6a. It shows an ordered molecular island adjacent to clean graphene, which exhibits a moiré pattern (inset to Figure 6a). The unit cell of the regular molecular superstructure can be inferred from the close-up view in Figure 6b. The lattice vectors are **a** and **b** with 
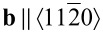
 (crystallographic direction of the graphene lattice, marked by the dashed line in Figure 6b), |**a**| = 1.40 ± 0.10 nm, |**b**| = 1.70 ± 0.10 nm and 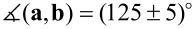. While the unit cell geometry is nearly identical to the one of C_42_H_28_ on Au(111), the surface density of C_42_H_28_ on graphene is approx. 0.51 nm^−2^ and, thus, lower than that observed on Au(111). The lower molecule density may tentatively be explained by a molecular superstructure that is formed owing to the optimization of the intermolecular coupling. This might be facilitated by very low energy barriers, which are even lower than those observed for C_42_H_28_ on Au(111), for translational and rotational degrees of freedom. A template effect of graphene or a moiré lattice on the molecular assembly was not identified. Merely the orientation of the tetracene backbone perpendicular to a 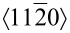 graphene crystallographic direction was observed. A finite residual coupling of the molecule to graphene-covered Pt(111) is likewise indicated by the comparison of the superstructure in Figure 6 with the one obtained for a thicker C_42_H_28_ film on graphene-covered SiC(0001) [45]. In the latter work a smaller unit cell was reported with individual C_42_H_28_ molecules appearing uniformly in STM images. It is likely that the difference to the superstructure depicted in Figure 6 results from an efficiently decoupled molecule residing on top of a molecular film deposited on nearly free graphene on SiC(0001).

Similar to observations on Pt(111), C_42_H_28_ molecules on graphene appear with two or three lobes in STM images, which are labeled α and β in Figure 6b. The two lobes at opposite sites of a dim separating line, which is assigned to the tetracene backbone, are attributed to the two phenyl groups, while the third lobe at one end of the dim line is caused by the tilting of the backbone. Therefore, α-C_42_H_28_ on graphene has an apparently planar tetracene moiety, while β-C_42_H_28_ exhibits a tilted and possibly twisted backbone. Very often, rows of β-C_42_H_28_ are observed within the compact C_42_H_28_ island (Figure 6c). Inside the row, pairs of β-molecules orient the upward tilted part of their tetracene backbone towards each other. The coexistence of different C_42_H_28_ configurations was previously reported for a variety of surfaces [25,35–36] and is not specific to graphene.

**Figure 7:**
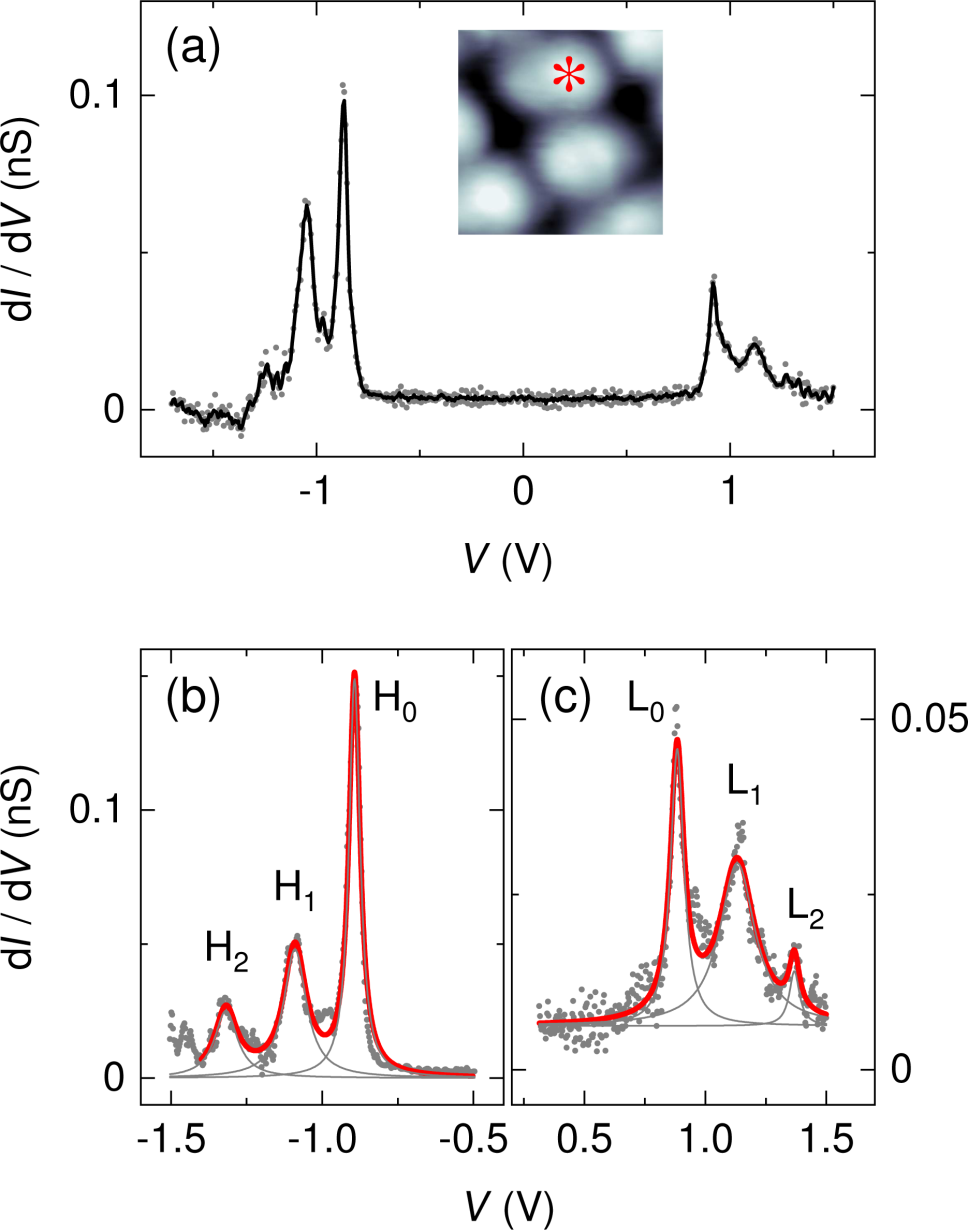
(a) Spectrum of d*I*/d*V* (dots) recorded above a C_42_H_28_ phenyl group on graphene-covered Pt(111) (feedback loop parameters: −1.5 V, 15 pA). The solid line represents smoothed data. Inset: STM image of a single C_42_H_28_ molecule on graphene-covered Pt(111) (2 V, 20 pA, 2 × 2 nm^2^) with an asterisk marking the position of spectroscopy. (b) Close-up d*I*/d*V* spectrum (dots) of the HOMO (H_0_) spectral fine structure (H_1_, H_2_) (feedback loop parameters: −1.5 V, 30 pA). The thick solid line represents a fit of three Lorentzian line shapes (thin gray lines) and a constant background to the data. (c) Like (b), for the LUMO (L_0_) with vibronic fine structure (L_1_, L_2_) (feedback loop parameters: 1.5 V, 20 pA).

Spectroscopy data corroborate the weak hybridization of C_42_H_28_ with the graphene-covered Pt(111) surface. Figure 7a shows a spectrum of d*I*/d*V* acquired atop a C_42_H_28_ molecule embedded in a molecular island. The spectroscopic signatures of HOMO and LUMO are visible at, respectively, approx. −0.87 and approx. 0.92 V. The molecular resonance widths are smaller than those observed on the other surfaces; in particular, the LUMO exhibits a narrow line shape.

Before analyzing the vibronic subbands of the molecular resonances we remark that the HOMO–LUMO gap width defined as the difference between LUMO and HOMO energy varies between approx. 1.6 eV and approx. 1.8 eV, depending on the individual molecule. The approx. 0.2 eV variation of the gap width is due to changes in the LUMO energy since the HOMO resonance essentially retains its energy at approx. −0.87 eV. Assuming that C_42_H_28_ is weakly coupled to the substrate, its orbital energies are expected to be aligned with the vacuum level [46] and, thus, susceptible to local changes in the work function. Site-specific work functions are indeed present on the moiré lattice of graphene. For instance, graphene-covered Ir(111), which may serve as a reference for graphene on Pt(111) owing to the comparably low hybridization of graphene with the two metal surfaces, exhibits work function changes of the order of 0.1 eV [47]. In the experiments presented here, the LUMO experiences changes in its energy while the HOMO is essentially pinned, which at first sight contradicts the alignment of the orbitals with the vacuum level. However, C_42_H_28_ is an electron donor and has the propensity to transfer negative charge to the substrate, which could in principle reduce or compensate the work-function-induced shift of the HOMO. A similar behavior was previously reported for C_64_H_36_ adsorbed on h-BN-covered Ru(0001) [48]. Intermolecular couplings [49] that were previously shown to induce strong orbital energy shifts due to different local molecular environments are unlikely to cause the LUMO energy variation because the molecular superstructure in the present case is regular. Moreover, a shift of the LUMO peak due to the electric field between tip and surface [50–51] can likewise be excluded due to the magnitude of the effect and the similar tip–molecule distances in the experiment.

Vibronic fine structure is visible in the two frontier orbitals giving rise to vibronic subbands of the HOMO, H_1_ and H_2_, with equidistant spacings of 180 ± 10 mV, and for the LUMO, L_1_ and L_2_, with equidistant spacings of 220 ± 10 mV. In order to clearly see the vibronic transitions in the HOMO and LUMO spectroscopic signatures, close-up views with increased bias voltage sampling are presented in Figure 7b for the HOMO and in Figure 7c for the LUMO. The raw d*I*/d*V* data were fit by the superposition of three individual Lorentzian line shapes (thin lines) on a constant background. Therefore, different groups of vibrational modes participate in the vibronic progression of the different frontier orbitals. Within the uncertainty margin the vibrational quantum with energy *h*ν_1_ ≈ 180 meV is most likely the same mode as that observed for C_42_H_28_ on Au(111) and corresponds to the vibrational mode with large Holstein coupling (vide supra). The second vibrational quantum with energy *h*ν_2_ ≈ 220 meV is likely to coincide with the C_42_H_28_ vibration with energy 1594 cm^−1^ ≈ 200 meV, which was likewise predicted to exhibit an elevated Holstein coupling [42]. The calculations [42] were performed for the HOMO alone. However, both vibronic excitations mainly involve tetracene stretching vibrations and both the HOMO and the LUMO are close to the tetracene backbone [43–44]. Therefore, vibronic progression induced by one of these vibrational modes is likely to occur in the LUMO as well. The exact displacement patterns of the modes at 1349 cm^−1^ and 1594 cm^−1^ differ [42]. The different symmetries of the displacement patterns may explain why the 1349 cm^−1^ (1594 cm^−1^) mode couples particularly well to the HOMO (LUMO). It has recently been shown that matching symmetries of vibrational and electronic states are favorable for the occurrence of vibronic progression [9]. Using Equation 3, the Huang–Rhys factor for vibronic progression of the HOMO (LUMO) is approx. 0.4 (approx. 0.6).

### Comparison

Topographic and spectroscopic data of C_42_H_28_ on Pt(111), Au(111) and graphene-covered Pt(111) consistently show the progressive reduction of the molecule–substrate interaction. The hit-and-stick adsorption of C_42_H_28_ on Pt(111) indicates a strong suppression of the C_42_H_28_ mobility after adsorption. Molecular orbitals leave weak and broad signatures in d*I*/d*V* spectra.

Using the same preparation parameters for C_42_H_28_ deposition on Au(111) as on Pt(111) leads to island growth with a regular superstructure. The crystalline adsorption phase unravels that low energy barriers for translation and rotation of the molecule exist because the individual C_42_H_28_ molecules can optimize the coupling to adjacent molecules. A finite adsorbate–substrate interaction is reflected by the presence of a molecular superstructure that matches the period of the Au(111) reconstruction. However, the HOMO resonance width has decreased by a factor of three compared to its width on Pt(111). Even vibronic progression due to a group of molecular vibrations with an energy of approx. 160 meV and a Huang–Rhys factor of approx. 0.5 is visible in the HOMO spectral line shape, which for molecules adsorbed on metal surfaces is exceptional. Unoccupied molecular orbitals, in contrast, are weak and broad with no indication of vibronic progression.

The most effective reduction of the C_42_H_28_–metal hybridization is achieved by adsorption of C_42_H_28_ on graphene-covered Pt(111). The unit cell of the molecular superstructure is similar to the one observed on Au(111), albeit with a lower molecule surface density and with no additional moiré pattern. Compared to direct adsorption on Pt(111) the HOMO resonance width of C_42_H_28_ on graphene-covered Pt(111) is reduced by more than a factor of ten. Likewise, the LUMO appears as a sharply peaked signature in d*I*/d*V* spectroscopy. Different groups of C_42_H_28_ vibrational quanta induce vibronic progression of the frontier orbitals. The HOMO exhibits vibronic fine structure due to vibrations with *h*ν_1_ ≈ 180 meV and a Huang–Rhys factor of 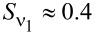, while the LUMO shows vibronic side bands due to molecular vibrational quanta with *h*ν_2_ ≈ 220 meV and 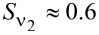.

## Conclusion

Using the hydrocarbon molecule C_42_H_28_ the progressive reduction of the molecule–surface hybridization can be achieved by using inert metal substrates, Au rather than Pt, and by introducing a two-dimensional material, e.g., graphene, as a buffer layer between the adsorbed molecule and the metal. The degree of decoupling can be judged by the molecule assembly after adsorption, the spectral line width of molecular resonances and the occurrence of vibronic progression. The most effective reduction of residual C_42_H_28_–metal hybridization was achieved here by adsorption on graphene-covered Pt(111). On this surface C_42_H_28_ exhibits vibronic progression in both frontier orbitals, induced by different molecular vibrational quanta and with different Huang–Rhys factors. Consequently, graphene represents an appropriate buffer layer for exploring electronic and vibronic properties at the single-molecule level.
